# Topics of Public Interest

**Published:** 1906-01

**Authors:** 


### TOPICS OF PUBLIC INTEREST.

### The President Gives Medal.

CAPTAIN CHURCH'S GALLANTRY RECOGNISED — NEEDS OF THE MEDICAL CORPS.

[*N. Y. Tribune*, Jan. 11, 1906.]

ONE of the heroes of the battle of Las Guasimas, Cuba, in the Spanish-American War, was rewarded recently by the presentation by the President of a medal of honor conferred by Congress for conspicuous gallantry in action. The recepient of the medal was Captain James Robb Church, of the medical corps of the army, who was a first lieutenant and assistant surgeon in the Rough Riders.

The presentation was made the occasion, on account of the notable character of Captain Church's services, of a brilliant ceremony. In the presence of a distinguished assemblage, including Secretary Taft, Lieutenant-General Chaffee, and members of the General Staff of the army and several officers of the navy, including Surgeon-General Rixey, all in full dress uniform. and members of the legislative council of the American Medical Association, President Roosevelt presented the medal to Captain Church. The ceremony took place in the President's private office and the adjoining Cabinet room. Ranged around the office were members of the General Staff and other uniformed officers, and in the Cabinet room were the members of the Medical Association and invited guests.

Secretary Taft formally presented Captain Church to the President, saying he had been recommended for a medal of honor for distinguished gallantry and service other than that directly called for in the line of his duty. The secretary called attention to the fact that General Leonard Wood, then colonel of the Rough Riders, and the President himself, both as lieutenant-colonel and colonel of the regiment, had recommended that the medal be conferred upon Captain Church. The secretary then read from the official records, quoting both general Wood and the President. The former reported to the War Department:

In the heat of the action Dr. Church came on the firing line repeatedly, and in addition to giving the men the necessary surgical assistance took some five of them on his back and carried them under a very heavy fire and with greatest exposure to himself to the rear. His services might have been legitimately considered ended for the time being, in giving them the best possible surgical aid and such assistance as he could render on the spot; but in addition to this he performed the duty above specified, carrying the men several hundred yards over an elevated ridge in the open, displaying in so doing the greatest gallantry and disregard for his own life in his efforts to remove the wounded men to a position of safety, where greater assistance could be furnished them.

Turning to Captain Church, President Roosevelt, holding in his hand the morocco case in which the medal was enclosed, addressed him as follows:

Captain Church: There is no distinction which confers greater honor on any American in military or civil life than this —the honor coveted above all others by every man in the military service of the United States. It was my good fortune, as colonel of the regiment in which you served, to be an eyewitness to your gallantry and to bear testimony to it by letter to the proper authorities, stating the reasons why I deemed that you were entitled to this medal of honor.

I wish to state, Mr. Secretary, that the letters I wrote were written before I was President. Since I was President I have held no communication whatsoever with the military authorities on the subject. Captain Church, there could be no greater pleasure than I now experience in handing to my old comrade and friend this medal of honor.

Grasping Captain Church's hand, the President remarked as an aside: "There is no greater comradeship than that which comes from having lived in the trenches together."

Captain Church bowed, but words evidently failed, and he accepted the medal in silence.

Turning then to the members of the American Medical Association, the President said with great earnestness:

I want to say just a word of greeting to you and to ask your influence on behalf of the medical corps, not only of the army, but of the navy. There is not a more exacting profession; there is not a profession which makes greater demands on those following it and which more entitles them to the gratitude of mankind than the profession which is yours. The army surgeon has to combine the work of your profession with the work of the military men of the line. In saying that I want to call your attention to two specific things—one thing that is now being done by men of your profession and one need of men of your profession.

First, the thing that is being done: All the United States is the debtor to the medical men who have accomplished such remarkable work on the Isthmus of Panama. You hear very loose talk about making the dirt fly in Panama. Before making the dirt fly it was necessary to get the microbes under; it was necessary to grapple with the mosquitoes; necessary to eradicate disease. That has been done to perfection. We have had the foundation laid for that wonderful piece of constructive engineering work, to dig the giant canal. Too much praise cannot be given to those who have done this work in Panama.

So much for tribute to your compeers. Now as to the need of your compeers. You recollect the complaint made about hygienic conditions during the war with Spain. Complaint was made that the troops were not properly treated, etc. The blame rested not on any man then in office, but upon our people as a whole, who had declined, through their representatives, to make provision long an advance for meeting such a need. If we had a war break out to-morrow and had to raise any large army, there would be an immediate breakdown in the medical department, simply because at present our medical corps is numerically only fit to take care of about 40 per cent of the regular army as it is now. The medical corps is not numerically fit to graple with a campaign in which our whole army as it is, the little army as it is, should be employed. And, of course, if we had to mobilize an army of volunteers we would, under present conditions, have to count upon widespread disaster through the shortcomings in the medical and sanitary and hygienic arrangements rendered inevitable by our present lack of preparation.

The Japanese have given us a good lesson in this, as in many other particulars, by the way they handled their army in the recent war. One of the reasons why their medical departmnt did well—the main reason—was the fact that they had an ample supply of doctors who had been practised in time of peace in doing the duties they would have to do in war. And until we have provision for an ample corps of doctors in the army, so that they can be practised in time of peace, we will not have prepared as we ought to prepare for the possibilities of war. Until we thus prepare we can make up our minds that we are ourselves responsible for any disaster that occurs to any army that the United States may raise in the future; not the man who may be at the head of the army at the time. The tendency is to attack the men in office at the time. That is utterly unjust, and the people themselves and the representatives of the people in public life who have failed to provide the necessary means in advance, they are responsible when disaster comes. That applies to the medical department, and it applies to every other branch of the military establishment just as much.

At the close of his remarks President Roosevelt was presented by Captain Church to his wife and some other relatives who were present. Later, Captain Church was a guest of the President at luncheon.

---

Doctor (to Mrs. Perkins. whose husband is ill)—Has he had any lucid intervals?

Mrs. Perkins (with dignity)—'E's 'ad nothing except what you ordered, doctor—(*Kansas City Independent*).

